# Acute respiratory distress syndrome: analysis of incidence and mortality in a university hospital critical care unit

**DOI:** 10.1186/cc11003

**Published:** 2012-03-20

**Authors:** JF Figueira, MO Oliveros, JL López, BC Civantos, LF Fernández

**Affiliations:** 1 Hospital Universitario La Paz, Madrid, Spain

## Introduction

The aim was to determine the incidence of acute respiratory distress syndrome (ARDS) in patients admitted to a university hospital ICU, analyse the ICU and the in-hospital mortality, and evaluate the associated factors.

## Methods

A prospective study in an ICU from October 2008 to January 2011. The ICU comprises 20 beds in a medical-surgical area, 10 in a critical burns area. All patients who underwent mechanical ventilation (MV) during 48 hours or more and who fulfilled ARDS criteria as defined by the 1994 American-European Consensus Conference on ARDS were included. All patients were ventilated following the protective MV strategy recommended.

## Results

During this period 1,900 patients were admitted, 697 needed MV for at least 48 hours and 108 fulfilled the ARDS criteria (5.6% of those admitted, 17% of the group on MV); 63% were male. The patients' age was 52 ± 12. The APACHE II score on admission was 23 ± 7, in survivors (S) 20 ± 7 and 24 ± 6 in nonsurvivors (NS) (*P *= 0.002). ARDS was primary in 70% and secondary in 30%. The most common aetiology was pneumonia (53%) followed by sepsis of intra-abdominal origin (15%). Duration of MV was 32.7 ± 30.2 days in S, 20.79 ± 20.73 in NS (*P *= 0.019). Survivors' mean length of stay was 35 ± 24 days, 23 ± 20 for NS (*P *= 0.007). ICU mortality was 49% and in-hospital mortality was 55%. Primary ARDS had an ICU mortality of 47%, an in-hospital mortality of 52%. Secondary ARDS had a 55% ICU mortality, an in-hospital mortality of 64%. Duration of primary ARDS was longer, 15.3 ± 12.2 versus 8.7 ± 79. Globally the main cause of death was multiple organ dysfunction, predominantly respiratory failure (55%). In primary ARDS the main cause of death was chiefly pulmonary (69%), while in secondary ARDS it was mainly multiple organ dysfunction associated with septic shock (71%). Factors associated with increased mortality were APACHE II score >23 and the presence of multiple organ dysfunction.

## Conclusion

Certain controversy remains regarding a decrease in ARDS-related mortality. Despite the fact that its incidence is not very high, it is still a clinical entity with a high mortality, and with a prognosis influenced not only by the degree of pulmonary involvement but by the association with multiple organ dysfunction.
